# Postgraduate corner: Continuing medical education: Psychopharmacology

**DOI:** 10.4103/0019-5545.55096

**Published:** 2009

**Authors:** Chittaranjan Andrade

**Affiliations:** Department of Psychopharmacology, National Institute of Mental Health and Neurosciences, Bangalore - 560 029, India

## CME QUESTIONS

A) Fibromyalgia is a poorly understod syndrome that is characterized by chronic, widespread musculoskeletal and joint pain. There are often multiple tender nodules present in specific anatomical loci, such as over the shoulders and back. Common accompanying symptoms include stiffness, fatigue, mood disturbance, and insomnia. Affected patients suffer from pain-related disability and impaired functioning in everyday life. With this background, mark *True* or *False* against each of the following statements:

1. Escitalopram is an approved treatment for fibromyalgia.
2. Duloxetine 120mg/day is more effective than duloxetine 60mg/day in the treatment of fibromyalgia.
3. An adequate trial of duloxetine for fibromyalgia should last at least 4-6 months.
4. In addition to attenuating measures of pain, duloxetine improves functional outcomes in fibromyalgia.
5. Fibromyalgia treatments have demonstrated efficacy in long-term as well as short-term management.

B) Tamoxifen is prescribed for various indications to women with estrogen receptor-positive breast cancer. These women may require antidepressant medication for concurrent depression; or for the treatment of hot flushes and other symptoms of artificial menopause. With this background, mark *True* or *False* against each of the following statements:

1. Tamoxifen is metabolized by CYP3A4.
2. Mirtazapine should be preferred to duloxetine over depressed women who are receiving tamoxifen.

C) Benzodiazepines have anxiolytic and anticonvulsant properties and are cross-tolerant with alcohol. They are therefore prescribed during alcohol withdrawal to reduce the severity of the withdrawal syndrome and to reduce the risk of withdrawal seizures. Diazepam and chlordiazepoxide are traditionally preferred for this indication because both have long half-lives of approximately 1-2 days, each; their levels in blood therefore tend to remain uniform across the course of the day, and drug withdrawal difficulties are fewer. However, they are metabolized by demethylation and hydroxylation and have active metabolites; as a result of these pharmacokinetic properties, they may accumulate, and mask or even precipitate hepatic encephalopathy in patients with alcoholic liver disease. With this background, mark *True* or *False* against each of the following statements:

1. For important theoretical reasons, if alcoholic liver disease is present or suspected, lorazepam should be avoided in patients who are being withdrawn from alcohol.
2. Clinical data exist to suggest that lorazepam may be effective in the management of uncomplicated alcohol withdrawal.

## CME ANSWERS

- **Fibromyalgia**
  Answers: 1. False; 2. False; 3. False. 4. True; 5. True.
  1. Citalopram appears ineffective in patients with fibromyalgia[1,2] and, to judge from a PubMed search conducted on June 14, 2009, escitalopram has not been studied for this indication. Pregabalin,[3,4] duloxetine[5,6] and milnacipran[7,8] are USA Food and Drug Administration-approved treatments for fibromyalgia. However, as serotonergic and noradrenergic mechanisms have both been described to mediate pain relief,[9] it is likely that most antidepressants, especially dual-acting drugs such as the tricyclic antidepressants, could be effective treatments for fibromyalgia. In other words, antidepressant efficacy against fibromyalgia could be a class action.
  2. Short-term, randomized, double-blind, placebo-controlled clinical trials of duloxetine (60 or 120mg/day) in patients with fibromyalgia found that the 120mg/day dose was not better than the 60mg/day dose.[10,11] A 1-year randomized controlled study also found no advantage with the higher dose.[12] For the majority of patients, therefore, 60mg/day is likely an adequate dose; whereas some patients may indeed require a higher dose, it should be kept in mind that the higher dose could increase the adverse effect burden.[12] With milnacipran, however, there are some small advantages with the 200mg/day dose relative to the 100mg/day dose in patients with fibromyalgia.[7,8] With pregabalin, too, a dose response relationship may exist between 300mg/day and 600mg/day.[3]
  3. In a study of the long-term safety and efficacy of duloxetine for fibromyalgia, Chappell *et al.*,[12] treated 350 patients with duloxetine 60mg/day for 8 weeks. Afterward, patients were randomized to continue on the same dose or receive 120mg/day for 1 year. There were significant improvements in pain severity, disability, and other outcome measures during the 8-week open label phase; 35% of patients met response criteria. In patients classified as nonresponders during the open label phase, improvements during the double-blind phase were similar irrespective of whether they received 60mg/day or 120mg/day, and the magnitude of additional improvement was small, indicating that the continuation of treatment beyond 8 weeks is unlikely to much improve outcomes in nonresponders. In other words, 8 weeks is likely an adequate trial duration when duloxetine is used to treat fibromyalgia.
  4. Duloxetine, milnacipran, and pregabalin all attenuate pain, reduce disability, and improve functional outcomes in patients with fibromyalgia.[3,7,12]
  5. Chappell *et al.*,[12] found that the 8-week efficacy of duloxetine in fibromyalgia was maintained across a 52-week continuation phase. Pregabalin[4] and milnacipran[13] have also shown maintained efficacy in the long term. Interestingly, some loss of efficacy across time was observed in a 6-month pregabalin trial[4] but not in the 52-week duloxetine trial;[12] this could have been related to the study design – the pregabalin trial[4] randomized open label responders to continue with pregabalin or switch to placebo, whereas the duloxetine trial[12] randomized open label completers to continue on the same dose of duloxetine or switch to a higher dose. Patient confidence could have been diminished by the placebo-controlled design in the pregabalin study,[4] but would not have been impacted in the duloxetine study.[12]
- **Antidepressant interactions with tamoxifen**
  Answers: 1. False; 2. True.
  1. Tamoxifen is converted by CYP2D6 into several active metabolites, prominent among which are 4-OH-tamoxifen and endoxifen. Persons who are 2D6 poor metabolizers have lower levels of endoxifen; some (but not all) studies show that such patients do less well with tamoxifen therapy.[14,15]
  2. Antidepressants such as fluoxetine, paroxetine, bupropion, and duloxetine inhibit CYP2D6; fluvoxamine, sertraline, and venlafaxine also inhibit 2D6, though to a far lesser extent. These antidepressants could be expected to lower endoxifen levels, thereby potentially reducing the efficacy of tamoxifen.[14] In contrast, drugs such as mirtazapine, escitalopram, and milnacipran have no effect on 2D6 and can therefore be safely co-administered with tamoxifen. The interaction between 2D6 inhibitors and tamoxifen is clinically significant: a study on about 1300 women found that tamoxifen-treated women who received paroxetine, fluoxetine, or sertraline for at least a year had a 13.9% risk of recurrence of breast cancer in contrast with a recurrence rate of just 7.5% in women not receiving these drugs.[16]
- **Lorazepam for uncomplicated alcohol withdrawal**
  Answers: 1. False; 2. True.
  1. Lorazepam is a benzodiazepine with an intermediate half-life of 8-18 h. Lorazepam is metabolized by glucuronidation, a pathway that is relatively spared in early liver disease. Lorazepam has no active metabolites and is eliminated by the kidneys. Lorazepam is also a potent anxiolytic and anticonvulsant. These properties suggest that lorazepam could be a preferred benzodiazepine during alcohol withdrawal, especially in patients with liver disease.[17]
  2. Small studies with samples of around 50 patients each found that alcohol withdrawal complications such as delirium or seizures tended to be more frequent in lorazepam-treated patients than in those receiving diazepam or chlordiazepoxide. A limitation of these studies is that all used low starting doses of lorazepam (e.g. 6mg/day) and tapered and withdrew the drug across just 5 days.[18–21] In contrast, in a large (n=100), 12-day, randomized, double-blind study of the safety and efficacy of lorazepam vs chlordiazepoxide, Kumar *et al.*,[17] found that lorazepam (8mg/day in three divided doses, tapered and withdrawn across 8 days) was as good as chlordiazepoxide (80mg/day in three divided doses, tapered and withdrawn across 8 days) in patients with moderately severe, uncomplicated alcohol withdrawal. For the theoretical reasons referred to earlier, the findings of this study encourage the preference for lorazepam over diazepam or chlordiazepoxide for the management of alcohol withdrawal in patients with known or suspected alcoholic liver disease.
